# Supplementary material for: Definition of phosphatidylinositol 4,5-bisphosphate distribution by freeze-fracture replica labeling
Source: J Cell Biol. 2024 Nov 4;224(1):e202311067. doi: 10.1083/jcb.202311067 (PMC11535894; doi:10.1083/jcb.202311067)
Supplement: Table S1 — shows yeast strains used in this study. [file JCB_202311067_TableS1.doc]

**Supplementary Table 1.** Yeast strains used in this study.

| Name | Genotype | Reference/Origin |
| --- | --- | --- |
| SEY6210 | *MATα leu2-3,112 ura3-52 his3-Δ200 trp1-Δ901 suc2-Δ9 lys2-801* | Robinson et al, 1988 |
| YT270 | SEY6210; SEY6210; PMA1-yeGFP::TRP1 | This study |
| YT343 | SEY6210; SEY6210; SUR7-yeGFP::TRP1 | This study |
| YT 637 | SEY6210 harboring pRS314GPD-GFP-PLCδ1-2xPH | This study |
